# Gene Network Disruptions and Neurogenesis Defects in the Adult Ts1Cje Mouse Model of Down Syndrome

**DOI:** 10.1371/journal.pone.0011561

**Published:** 2010-07-16

**Authors:** Chelsee A. Hewitt, King-Hwa Ling, Tobias D. Merson, Ken M. Simpson, Matthew E. Ritchie, Sarah L. King, Melanie A. Pritchard, Gordon K. Smyth, Tim Thomas, Hamish S. Scott, Anne K. Voss

**Affiliations:** 1 The Walter and Eliza Hall Institute of Medical Research, Melbourne, Australia; 2 Department of Medical Biology, University of Melbourne, Melbourne, Australia; 3 Department of Pathology, The Peter MacCallum Cancer Centre, Melbourne, Australia; 4 Department of Molecular Pathology, The Centre for Cancer Biology, The Institute of Medical and Veterinary Science and The Hanson Institute, SA Pathology, and The Adelaide Cancer Research Institute, School of Medicine, University of Adelaide, Adelaide, Australia; 5 Department of Biochemistry and Molecular Biology, Monash University, Victoria, Australia; 6 Department of Obstetrics and Gynaecology, Faculty of Medicine and Health Sciences, Universiti Putra Malaysia, Selangor, Malaysia; Health Canada, Canada

## Abstract

**Background:**

Down syndrome (DS) individuals suffer mental retardation with further cognitive decline and early onset Alzheimer's disease.

**Methodology/Principal Findings:**

To understand how trisomy 21 causes these neurological abnormalities we investigated changes in gene expression networks combined with a systematic cell lineage analysis of adult neurogenesis using the Ts1Cje mouse model of DS. We demonstrated down regulation of a number of key genes involved in proliferation and cell cycle progression including *Mcm7, Brca2*, *Prim1, Cenpo* and *Aurka* in trisomic neurospheres. We found that trisomy did not affect the number of adult neural stem cells but resulted in reduced numbers of neural progenitors and neuroblasts. Analysis of differentiating adult Ts1Cje neural progenitors showed a severe reduction in numbers of neurons produced with a tendency for less elaborate neurites, whilst the numbers of astrocytes was increased.

**Conclusions/Significance:**

We have shown that trisomy affects a number of elements of adult neurogenesis likely to result in a progressive pathogenesis and consequently providing the potential for the development of therapies to slow progression of, or even ameliorate the neuronal deficits suffered by DS individuals.

## Introduction

Down syndrome (DS) is a result of trisomy or partial trisomy of human chromosome 21 (HSA21) and has an incidence of 1 in approximately 1000 live births [1]. Individuals are affected by a large number of abnormalities that vary in gravity and involve most organ systems. The foremost and most debilitating trait of DS is mental retardation, which ranges from mild to severe and is followed by a loss of cognitive abilities in adulthood and the development of early onset Alzheimer's disease (AD) [2], [3], [4]. How trisomy 21 results in cognitive impairment remains unclear, however, associated structural brain abnormalities have been described. The DS brain is characterised by a reduction in overall size and a disproportionate reduction in size of particular regions due to hypocellularity. These regions include the hippocampal dentate gyrus and cerebellum in the pre-natal brain and the cerebral hemispheres, frontal lobe, hippocampus and cerebellum in the postnatal brain [5], [6], [7], [8], [9], [10], [11], [12], [13], [14], [15]. Other neural abnormalities identified include altered cortical lamination and consistent dendritic and synaptic abnormalities [16], [17], [18], [19], [20], [21], [22], [23], [24]. The neuropathological marks of AD (neuritic plaques containing amyloid β surrounded by degenerating nerve terminals and neurofibrillary tangles composed of aggregated hyperphosphorylated tau) are present in essentially all adult DS brains, however, it seems that not all individuals develop clinically recognised AD [25].

We utilised a mouse model to investigate how trisomy 21 might affect gene regulatory networks and adult neurogenesis. Mouse chromosome 16 (MMU16) has significant homology with HSA21 [26]. A number of mice trisomic for large segments of MMU16 or HSA21 (for summary see Text S1) or trisomic for single HSA21 genes have been created. The development of three models in particular, trisomic (Ts16) and partially trisomic (Ts65Dn and Ts1Cje) for MMU16 has greatly facilitated investigations of the central nervous system [26], [27], [28]. Despite showing some similarities to DS, the trisomic chromosome carried by Ts16 contains genes not orthologous to HSA21, furthermore these mice die *in utero* precluding analysis of the postnatal consequences of trisomy [29], [30], [31].

A number of features analogous to DS have been demonfstrated in the partially trisomic mouse models and the extent and severity of the phenotypes observed mostly correlate with the size of the trisomic segment. Both Ts65Dn and Ts1Cje mice display craniofacial dysmorphology that directly parallels human DS and defective spatial learning and memory [32], [33], [34], [35], [36], [37], [38], [39], [40]. Abnormalities in physical structure of particular areas of the brain also seen in human DS have been demonstrated in the Ts65Dn and Ts1Cje models, which include hypocellularity in hippocampal structures including the dentate gyrus in Ts65Dn mice [41]. Furthermore, it has been shown that there is a progressive degeneration and loss of cholinergic neurons, which are particularly vulnerable in AD, in the basal forebrain of Ts65Dn [42], [43].

Proliferation defects have been demonstrated in the hippocampal dentate gyrus and neocortical germinal matrix of DS, Ts16 and Ts65Dn foetuses, postnatal Ts65Dn mice and in the hippocampal dentate gyrus and cerebellum of adult Ts65Dn mice [41], [44], [45], [46], [47], [48], [49], [50], [51], [52], [53]. More recently, impaired neurogenesis has been described in the hippocampal dentate gyrus and the subventricular zone (SVZ) of the lateral ventricles of adult Ts1Cje and Ts2Cje (genetic equivalent of Ts65Dn) mice and also in the embryonic cortex of both models [54]. Additionally, a reduction in proliferation of neurospheres isolated from the neocortex of Ts1Cje embryos has also been depicted recently [55].

Neurogenesis occurs prenatally and postnatally but also throughout adulthood, the latter in predominantly two regions of the brain, the subgranular zone of the dentate gyrus and the SVZ lining the lateral ventricles. Neural stem cells (NSCs) residing in neurogenic regions have the ability to self-renew and differentiate into neurons, astrocytes and oligodendrocytes [56], [57]. They give rise to transit amplifying neural progenitors and subsequently to neuroblasts with limited proliferative capacity. Neuroblasts in turn differentiate into neurons. Newly formed cells in the SVZ migrate rostrally along the lateral walls of the lateral ventricles forming the rostral migratory stream (RMS) to the olfactory bulbs where they become interneurons [58], [59], [60].

Based on a number of *in vivo* and *ex vivo* experiments we show that Ts1Cje adult neurogenesis is reduced as a result of abnormal progenitor production and defects in cell lineage commitment during differentiation rather than defects in NSC development or homeostasis. Moreover, we identified specific regulators of cell cycle progression that are dysregulated in neural precursors isolated from the adult Ts1Cje SVZ. We hypothesise that these defects in adult neurogenesis confound pre-existing developmental defects and are likely to progressively contribute to problems in memory formation and possibly brain functions throughout adult life. We also expect that the defects will impair the ability of the DS brain to respond to neuronal loss and degeneration with repair.

## Results

### Genes involved in proliferation are disrupted in Ts1Cje adult neurospheres

In order to identify genes dysregulated in adult Ts1Cje neurogenesis we performed a whole-genome expression analysis using GeneChip® Mouse Genome 430 2.0 Arrays (Affymetrix, Santa Clara, USA) on 3 pairs of female Ts1Cje and disomic littermate control primary neural stem and progenitor cells after expansion for 7 days as neurosphere cultures. This data is freely accessible on the Gene Expression Omnibus website under the series accession number GSE17760.

**Figure 1:**
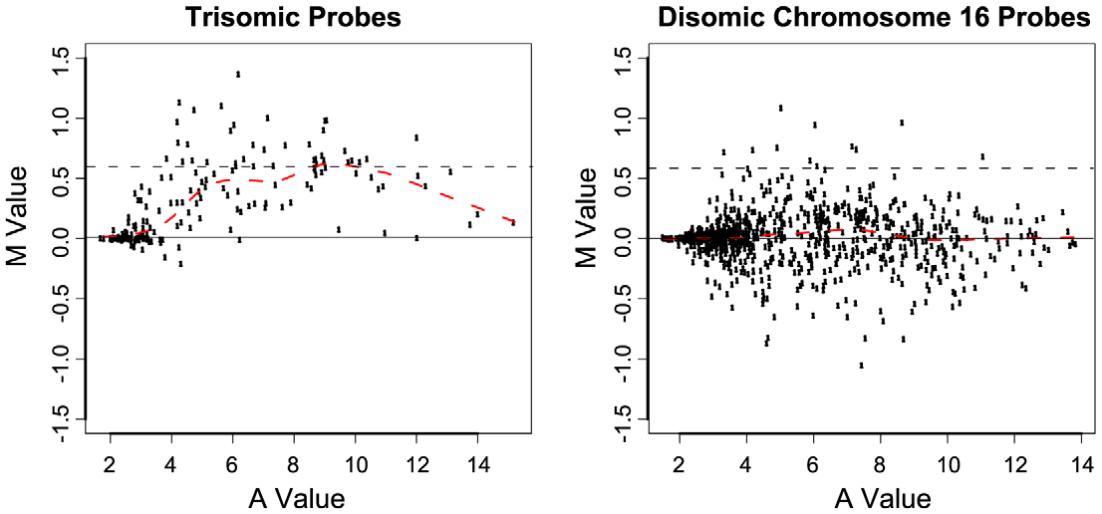
*MA* plots of MMU16 trisomic and disomic microarray probes support the gene dosage effect hypothesis. *M* is the average log_2_ fold change in intensity of fluorescence in the trisomic compared to the disomic (as a control). *A* is the average log_2_ intensity of fluorescence for both genotypes. The majority of expressed probes to genes located in the trisomic region had an *M* value of ∼0.58 =  fold change ∼1.5 (marked by a black dashed line), compared to the majority of disomic probes which have an *M* value of 0 =  fold change 1. The red dashed line is the line of loess fit to the *M* values.

To examine the overall behaviour of genes in the trisomic region, we plotted the log_2_ fold-changes (*M*) for trisomic versus disomic mice versus the average log_2_ expression (*A*). Each dot represents a single probe. Probes that were not expressed naturally did not show any change, i.e., the log_2_ fold-changes were around 0, but probes that were expressed at worthwhile levels (*A*-values >5) consistently showed fold changes of around 1.5 (equivalent to *M* value ∼0.58; Figure 1). Our data therefore supports the gene dosage effect hypothesis, which specifies that three copies of the majority of the genes involved in DS results in an increase in their expression of 50%.

Considering a trisomic gene copy number increase of only 0.5, no great changes in gene expression outside of the trisomic region are expected, which is what we observed in the gene expression profile of the Ts1Cje neurospheres. A global analysis of the *P*-values suggested that around 6.5% of expressed probes (2052 probes) may be differentially expressed, although the fold changes were small so that few genes could be individually identified as differentially expressed at conventional significance levels. In order to uncover changes in groups of functionally interconnected genes we utilised two online analysis tools the Database for Innovation, Visualization and Integrated Discovery (DAVID; [61] and Ingenuity Pathway Analysis (IPA).

**Figure 2:**
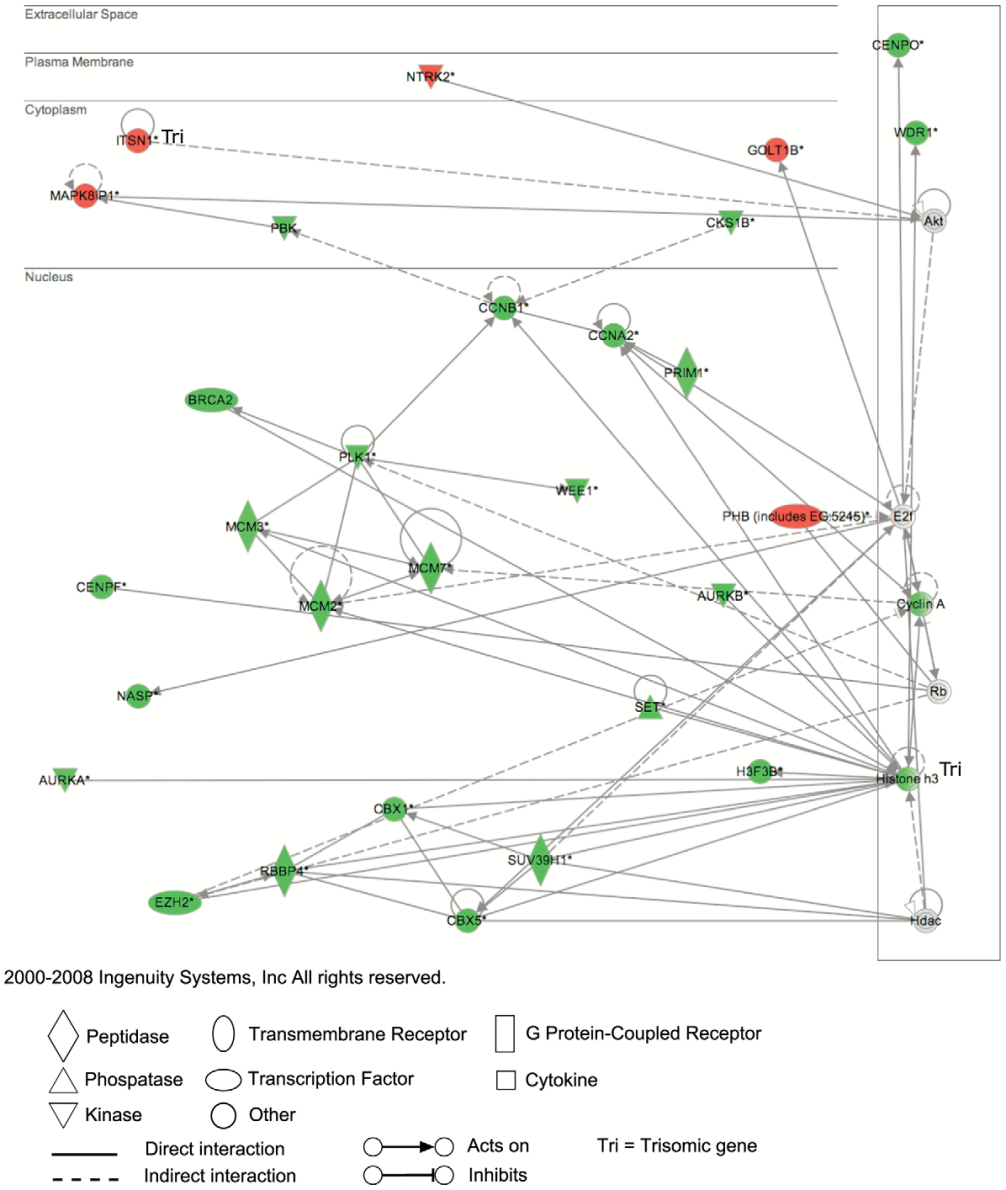
Gene network analysis of microarray data. IPA network containing the largest number of connected differentially expressed probes (cut off *t*-statistic ±4) from a comparison of 3 pairs of female Ts1Cje and disomic littermate control neurosphere cultures constructed from microarray expression data. The top functions associated with these probes include cell proliferation, DNA replication, recombination and repair, cell cycle and cancer. Green symbols represent probes that were downregulated in Ts1Cje neurospheres compared to disomic neurospheres, whilst red symbols represent probes that were upregulated in Ts1Cje neurospheres. Grey symbols represent connected probes that where unchanged in Ts1Cje neurospheres.

Intriguingly, the DAVID and IPA analyses highlighted that a significant number of genes that were differentially expressed were involved in proliferation with roles in cell cycle control, DNA replication, recombination and repair and chromosomal modifications (Table S1 and Figure 2). The majority of these genes were downregulated and disomic. Functional annotation of the probes that were over expressed in the Ts1Cje neuropsheres identified a significant enrichment associated with genes that are involved in general cellular functions including energy production, metabolism and endocytosis, suggesting that the cells may be under some degree of stress (Table S1).

**Figure 3:**
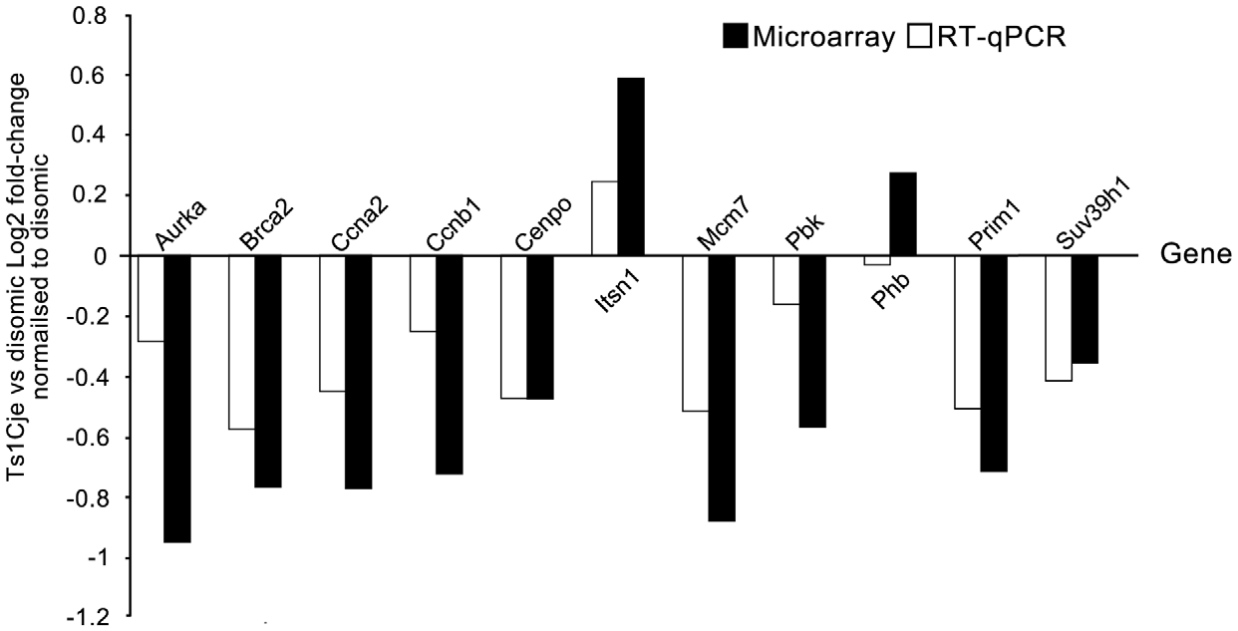
RT-qPCR and microarray neurosphere data for 11 selected proliferation associated genes. Microarray *M* values compared to the RT-qPCR Ts1Cje vs disomic log_2_ fold changes in expression normalised to the disomic neurospheres. RT-qPCR and microarray analysis were completed on 3 independent pairs of female 7-day-old Ts1Cje and disomic littermate control neurospheres. Both microarray and RT-qPCR differential expression was assessed using empirical Bayes moderated *t*-statistics [98] calculated using the limma package [100] and adjusted for multiple testing [99]. The trisomic gene *Itsn1* was upregulated in both the microarray and RT-qPCR data. All genes that were downregulated in the Ts1Cje compared to the disomic neurospheres by microarray analysis were also downregulated by RT-qPCR (*Aurka*, *Brca2*, *Ccna2*, *Ccnb1*, *Cenpo*, *Mcm7*, *Pbk*, *Prim1* and *Suv39h1*). *Phb* was upregulated in the Ts1Cje neurospheres by microarray analysis, but slightly downregulated by RT-qPCR.

Focusing on the IPA network with the largest number of differentially expressed probes (Figure 2) we analysed the expression of 11 genes (*Aurka*, *Brca2*, *Ccnb1*, *Ccna2*, *Cenpo*, *Itsn1* (trisomic), *Mcm7*, *Pbk*, *Phb*, *Prim1* and *Suv39h1*) by RT-qPCR in neurosphere cultures generated from 3 pairs of female Ts1Cje and disomic littermate controls independently of the original microarray analysis (Figure 3 and Table S2). Similar to the array results, the differences in expression between the genotypes were small but consistent. The direction of differential expression was validated in 10/11 genes. *Itsn1*, the trisomic gene was upregulated in the Ts1Cje neurospheres as expected. All genes that were downregulated in the Ts1Cje neurospheres by microarray analysis were also downregulated by RT-qPCR (*Aurka*, *Brca2*, *Ccnb1*, *Ccna2*, *Cenpo*, *Mcm7, Pbk, Prim1* and *Suv39h1*). *Phb* was shown to be slightly downregulated in the Ts1Cje neurospheres by RT-qPCR analysis but upregulated in the microarray analysis.

To examine whether these small abnormalities in gene expression affect the Ts1Cje brain we examined the neurogenic lineage systematically from neural stem cells (NSCs) through neural progenitors, via neuroblasts, to neuronal differentiation and neurite outgrowth. In addition, we also examined neuroblast migration.

### Normal numbers of neural stem cells in Ts1Cje *in vivo*

Gene expression abnormalities in proliferation associated genes imply that there are defects in the proliferation of trisomic NSCs and/or progenitors. We used a double labelling assay with the thymidine analogues 5-chloro-2′-deoxyuridine (CldU) and 5-iodo-2′-deoxyuridine (IdU) to investigate whether there is a reduction in the numbers of NSCs or a defect in the proliferation of NSCs in the adult Ts1Cje subventricular zone (SVZ). Thymidine analogues are incorporated into DNA during the DNA synthesis phase of the cell cycle and then diluted exponentially following cell division when half the analogue is distributed to the daughter cell. The thymidine analogue is undetectable by immunohistochemistry after 3 cell divisions.

NSCs are relatively quiescent with a relatively long cell cycle length of ∼15 days and an S phase of ∼8 h [62], [63]. In comparison, the proliferating proportion of the SVZ population of cells have an estimated cell cycle length of ∼12.7 h and an S phase of ∼4.2 h [64]. To investigate the thymidine analogue label retention of NSCs we injected 4 pairs of male Ts1Cje and disomic mice twice daily for 7 days with IdU, left them without treatment for 7 days, injected them twice daily with CldU for 7 days and then left them without treatment for 7 days. Based on the reported duration of cell cycles, S phases and thymidine analogue bioavailability of 2 h this labelling treatment strategy should label only NSCs as the thymidine label in neural progenitors will be diluted to levels below detection through a high number of cell divisions [65]. Neuroblasts that were in their last cycle division during thymidine labelling will have migrated away from the neurogenic zone to the olfactory bulbs.

**Figure 4:**
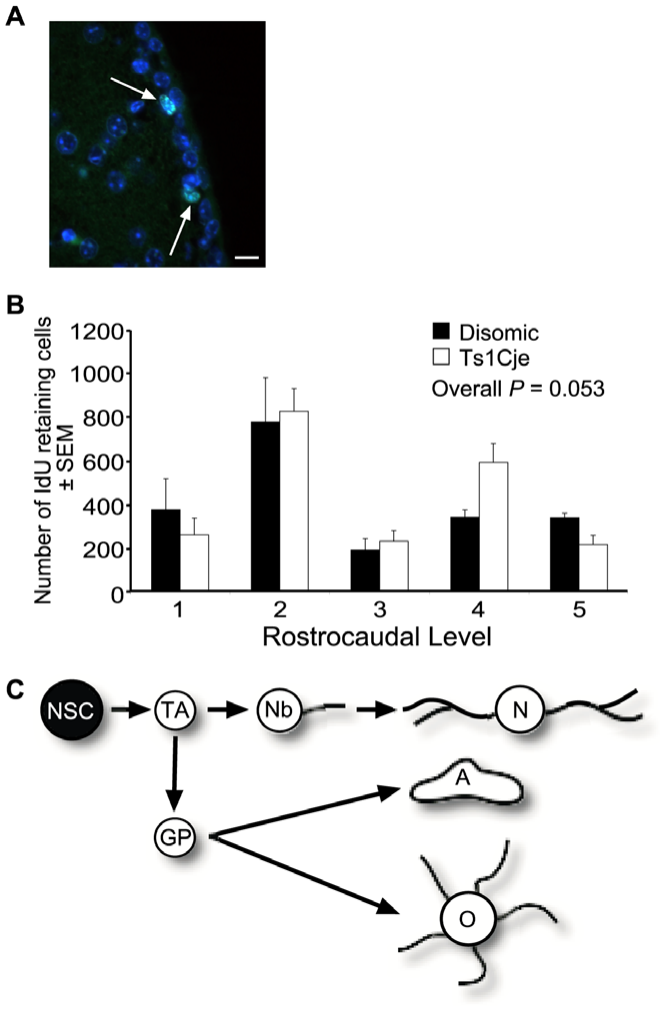
Trisomic neurogenic SVZ contain normal numbers of label retaining cells. (A) Immunofluorescence detection of typical IdU long-term retaining cells (marked by arrows), labelled with an anti-BrdU antibody, which also detected IdU but not CldU (clone B44, BD Biosciences; green) and DAPI nuclear counterstain (blue). Original magnification ×400. Scale bar  = 10 µm. (B) There was no difference in the number of IdU long-term retaining cells in the SVZ of Ts1Cje and disomic sex matched controls (*P* = 0.053; *N* = 4 pairs of male Ts1Cje and disomic littermate controls). The number of IdU retaining cells were counted at 5 equally spaced rostrocaudal levels. (C) Neural cell lineage schematic with the cell type examined highlighted – NSC, neural stem cell. Other cells: TA, transit amplifying cell; Nb, neuroblast; N, neuron; GP, glial precursor; A, astrocyte; O, oligodendrocyte.

IdU positive cells were counted in the subventricular neurogenic zone at 5 equally spaced rostrocaudal levels. We found no significant difference in the number of IdU long-term label retaining cells (*P* = 0.053; *N* = 4 pairs of male Ts1Cje and disomic controls; Figure 4), or double label (IdU and CldU) retaining cells (*P* = 0.97; *N* = 4 pairs of male Ts1Cje and disomic controls; data not shown). We therefore detected no difference in the number of NSCs in the adult Ts1Cje brain compared to their sex matched disomic littermate controls.

### Normal numbers of apoptotic cells in the Ts1Cje subventricular zone

**Figure 5:**
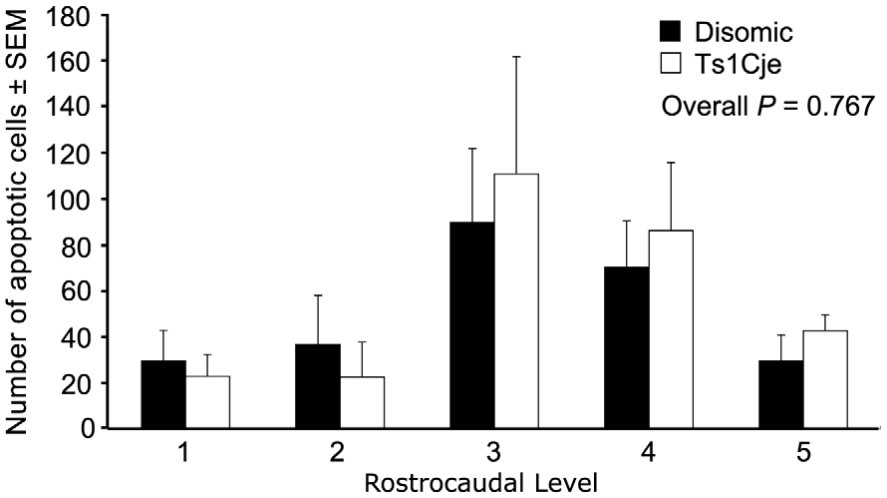
Assessment of cell death by apoptosis. There was no difference in the number of apoptotic cells in the SVZ of Ts1Cje and disomic sex matched controls (*P* = 0.767; *N* = 4 pairs of sex matched Ts1Cje and disomic littermate controls). Apoptotic cells were detected by TUNEL assay and counted at 5 equally spaced rostrocaudal levels.

We examined the incidence of cell death using the TUNEL assay. We found that there was no difference in the numbers of apoptotic cells in the SVZ between Ts1Cje and disomic mice demonstrating that the disruption in genes involved in proliferation have no affect on the rate of cell death (*P* = 0.767; *N* = 2 pairs of female and 2 pairs of male Ts1Cje and disomic controls; Figure 5). Given that our *in vivo* assay showed no difference in the number of NSCs in the Ts1Cje SVZ, we used an *ex vivo* assay to examine the self-renewal ability of adult NSCs and progenitors.

### Reduced number of neural progenitors in the Ts1Cje subventricular zone

**Figure 6:**
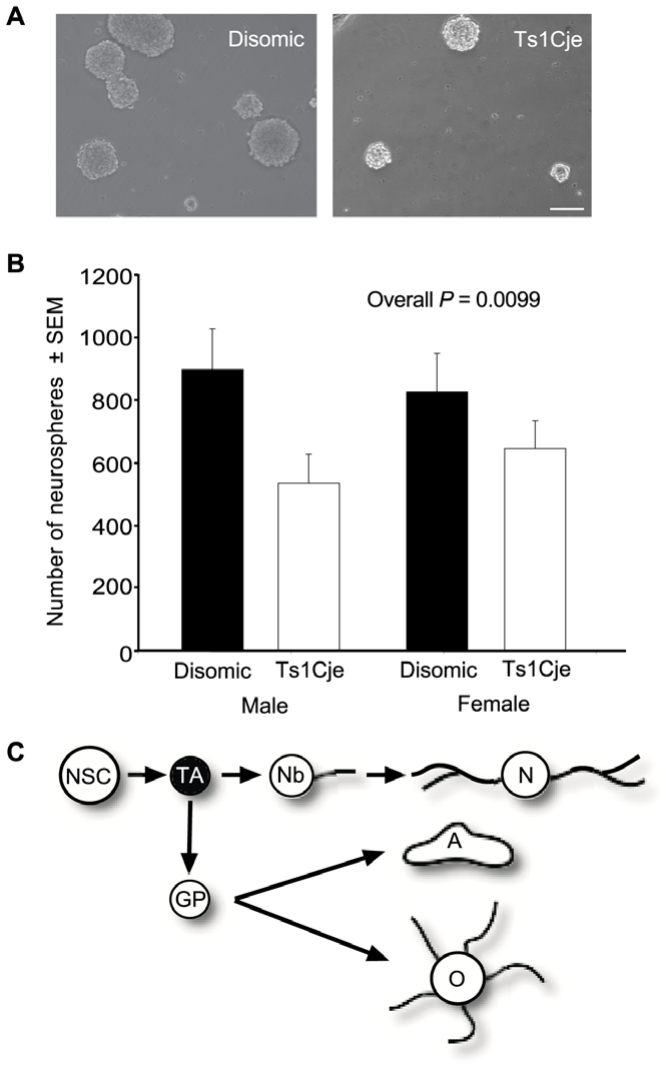
Trisomic SVZ contains fewer colony-forming cells. (A) Typical Ts1Cje and disomic neurosphere cultures. Original magnification ×20 Scale bar  = 100 µm. (B) The number of neurospheres derived from the SVZ of Ts1Cje mice was reduced compared to disomic sex matched littermate controls in both males and females (*P* = 0.0099; *N* = 4 pairs of female and 4 pairs of male Ts1Cje and disomic controls). (C) Neural cell lineage schematic with the cell type examined highlighted – TA, transit amplifying cell. Other cells: NSC, neural stem cell; Nb, neuroblast; N, neuron; GP, glial precursor; A, astrocyte; O, oligodendrocyte.

Adult NSCs and progenitors can be grown in proliferation medium containing EGF and FGF2 as floating colonies of cells or neurospheres [56], [66]. We utilised this characteristic to assess the ability of Ts1Cje adult NSCs and progenitors to give rise to colonies of undifferentiated cells. The number of neurospheres derived from the SVZ of Ts1Cje mice was consistently reduced, with an average 28% reduction compared to sex matched disomic littermate controls (*P* = 0.0099; *N* = 4 pairs of female and 4 pairs of male Ts1Cje and disomic controls; Figure 6A and B).

Since we found no difference in the number of long-term label retaining NSCs and the self-renewal ability of the stem cells is sufficient to maintain NSC numbers, the reduction in Ts1Cje neurosphere formation is most likely due to a reduced production of neural progenitor cells from NSCs. We subsequently examined whether the number of Ts1Cje neuroblasts was also reduced.

### Reduced numbers of migrating neuroblasts in the Ts1Cje rostral migratory stream

**Figure 7:**
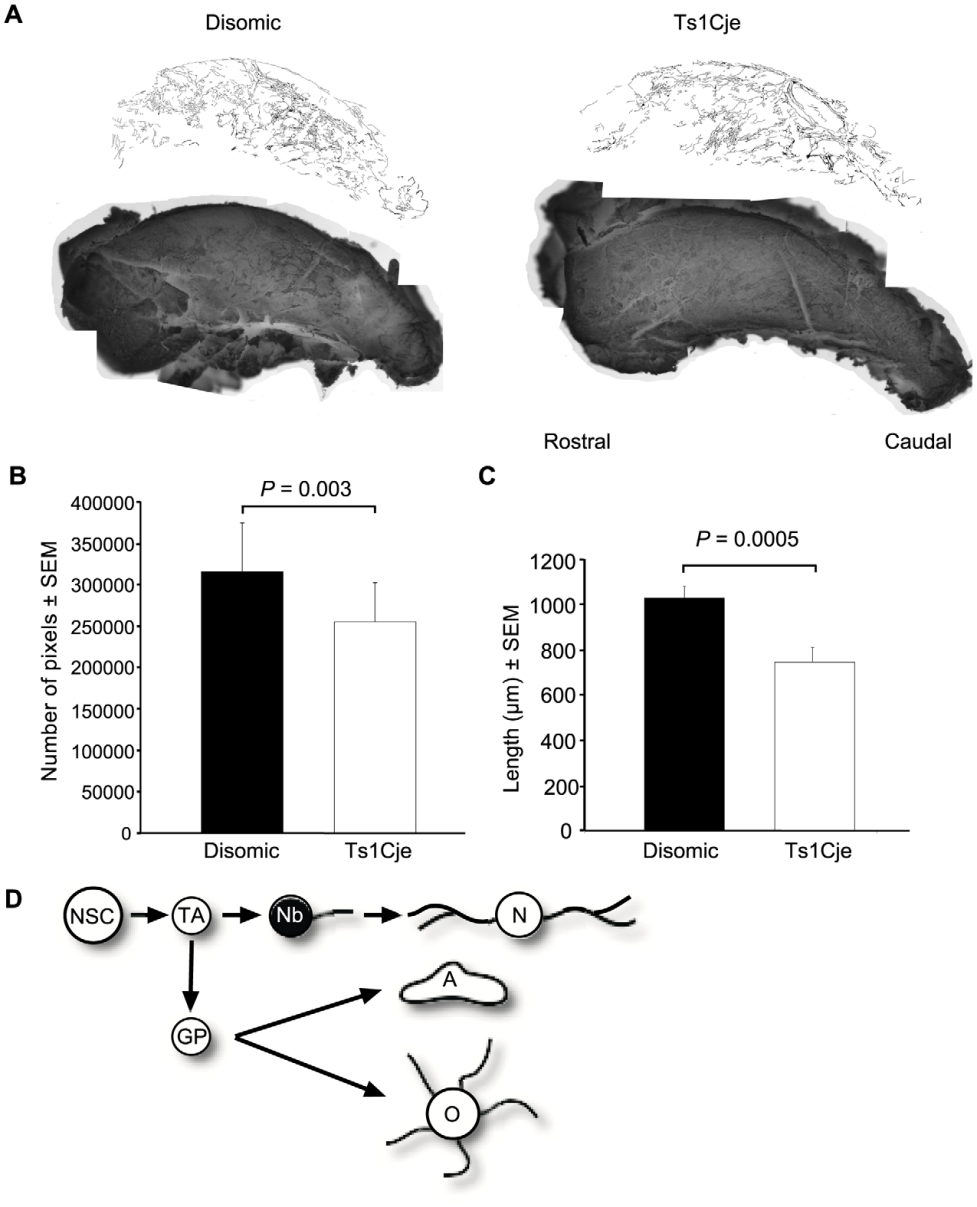
The Trisomic SVZ contains fewer migrating neuroblasts. (A) Whole mount immunohistochemistry of the lateral walls of the lateral ventricles of a typical adult Ts1Cje and disomic sex matched littermate pair, stained with an antibody against PSA-NCAM and the staining traced to allow quantification. (B) The traces of migratory neurons within the Ts1Cje RMS were reduced by an average of 18% compared to disomic controls (*P* = 0.003; *N* = 5 pairs of sex matched Ts1Cje and disomic littermate controls). (C) Measured using histological landmarks within the olfactory bulb, the anterior-posterior length of the Ts1Cje olfactory bulbs were reduced compared to disomic controls (*P* = 0.0005; *N* = 5 pairs of sex matched Ts1Cje and disomic littermate controls). (D) Neural cell lineage schematic with the cell type examined highlighted – Nb, neuroblast. Other cells: NSC, neural stem cell; TA, transit amplifying cell; N, neuron; GP, glial precursor; A, astrocyte; O, oligodendrocyte.

We quantified the size of the neuroblast population generated by the SVZs of Ts1Cje and disomic sex matched littermate controls. Neuroblasts express the cell surface molecule polysialic acid-neural cell adhesion molecule (PSA-NCAM) and move as chains of migrating neuroblasts from the SVZ to the olfactory bulbs where they become periglomerular and granule cell interneurons [58], [59], [60]. Migratory neuroblasts across the lateral walls of the lateral ventricles were labelled with an anti PSA-NCAM antibody, traced and quantified. In comparison to sex matched disomic controls, we identified a reduction in the absolute number of migratory neurons in Ts1Cje with an average of 18% (*P* = 0.003; *N* = 5 pairs of sex matched Ts1Cje and disomic littermate controls; Figure 7). Furthermore, through measurement of histological landmarks we show that the anterior-posterior length of the Ts1Cje olfactory bulbs are shorter than their disomic littermate controls (*P* = 0.0005; *N* = 5 pairs of sex matched Ts1Cje and disomic littermate controls; Figure 7).

The PSA-NCAM staining revealed that the Ts1Cje neuroblasts were reduced in number, but directional pattern of migration appeared normal and there was no obvious defects such as blockages or altered orientation. In the following experiment we examined their rate of migration using a medium-term thymidine analogue labelling assay.

### Tendency for the migration of Ts1Cje neuroblasts to be delayed

**Figure 8:**
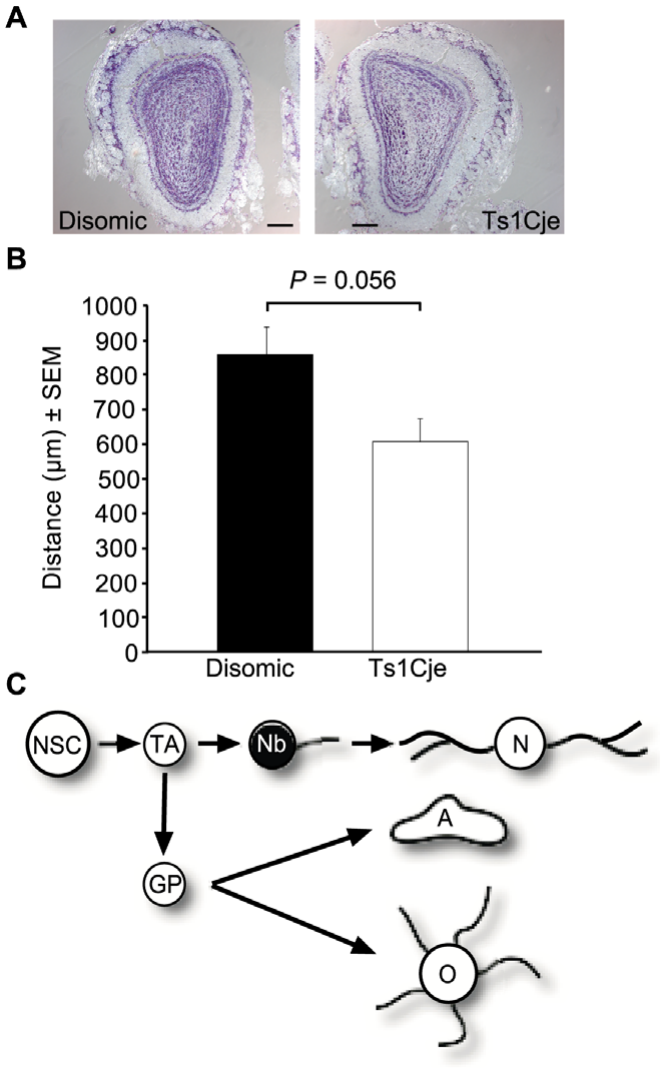
Assessment of neuroblast migration. (A) Cresyl violet stained sections adjacent to the migration front of short-term labelled BrdU stained neuroblasts (not shown) from a typical pair of Ts1Cje and disomic littermate controls. Comparison of histological landmarks highlighted a tendency of the trisomic neuroblasts to travel a shorter distance over the 6 days than disomic neuroblasts. Original magnification ×50. Scale bar = 200 µm. (B) The mean distance migrated into the olfactory bulb by Ts1Cje neuroblasts was on average 247.5 µm shorter than that migrated by disomic controls (*P = *0.056; *N* = 3 male and 1 female pairs of Ts1Cje and disomic littermates). (C) Neural cell lineage schematic with the cell type examined highlighted – Nb, neuroblast. Other cells: NSC, neural stem cell; TA, transit amplifying cell; N, neuron; GP, glial precursor; A, astrocyte; O, oligodendrocyte.

Neuroblasts take between 2 and 6 days to migrate from the SVZ to the olfactory bulbs via the RMS [58]. Mice were injected with the thymidine analogue 5-bromo-2′deoxyuridine (BrdU) twice daily for 4 days and left without treatment for 2 days. The migration front of the neuroblasts was identified in serial brain sections through positive staining for BrdU (Figure 8A). Following identification of the migratory front in the olfactory bulbs the distance from the frontal cortex to the migration front was measured (Figure 8B). In each pair of mice the disomic neuroblasts had migrated further into the olfactory bulbs. On average the labelled neuroblasts had migrated 247.5 µm further into the olfactory bulbs of disomic mice compared to Ts1Cje mice, however the difference was just below the level of significance due to large variations between animals (*P* = 0.056; *N* = 3 male and 1 female pairs of Ts1Cje and disomic littermates).

Finally, we examined the ultimate product of SVZ neurogenesis, the differentiating neuron.

### Reduced numbers of neurons arise from Ts1Cje neural progenitors

**Figure 9:**
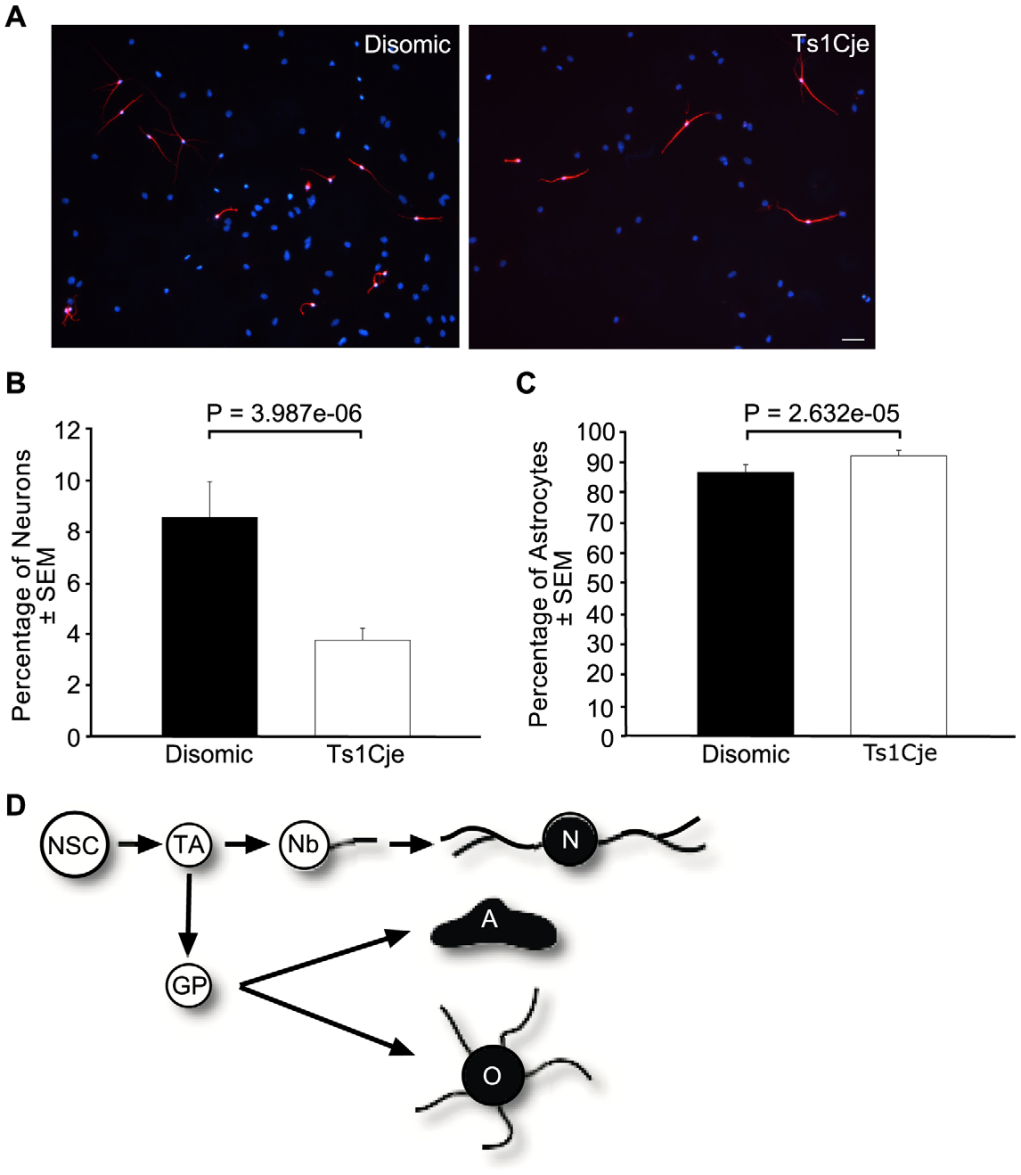
Trisomic neural progenitors form fewer neurons and more astrocytes. (A) Immunofluorescence of differentiated cells from dissociated neurospheres stained with an anti-β-tubulin type III antibody (red) labelling neurons and bisbenzimide nuclear counterstain (blue) (Original magnification ×400; Scale bar  = 20 µm). (B) The number of neurons generated from Ts1Cje neurosphere cultures was reduced compared to disomic neurosphere cultures with an average of 56% (*P* = 3.987e-06; *N* = 4 pairs of adult female Ts1Cje and disomic littermate controls). (C) The number of astrocytes generated from Ts1Cje neurosphere cultures was increased compared to disomic neurosphere cultures with an average of 6% (*P* = 2.632e-05; *N* = 4 pairs of adult female Ts1Cje and disomic littermate controls). (D) Neural cell lineage schematic with the cell type examined highlighted – N, neuron; A, astrocyte; O, oligodendrocyte. Other cells: NSC, neural stem cell; TA, transit amplifying cell; Nb, neuroblast; GP, glial precursor.

Removal of the growth mitogens EGF and FGF2 from neurosphere medium and addition of 1% foetal calf serum results in differentiation of NSCs and progenitors [56]. To determine whether Ts1Cje NSCs have a defect in neuronal differentiation we compared the number of neurons, astrocytes and oligodendrocytes produced by Ts1Cje and sex matched disomic littermate neurosphere cells *in vitro*. Following 7 days of primary culture, neurospheres were dissociated and plated onto poly-ornithine and laminin coated coverslips. Cell types were identified by immunofluorescence using antibodies against type III β-tubulin, glial fibrillary astrocyte protein (GFAP) and O4 to mark neurons, astrocytes and oligodendrocytes respectively. The number of neurons generated from Ts1Cje neurospheres was reduced with an average of 56% compared to sex matched disomic littermate controls (*P* = 3.987e-06; *N* = 4 pairs of adult female Ts1Cje and disomic littermate controls; Figure 9A). In contrast and correspondingly, the number of astrocytes generated from Ts1Cje neurosphere cells was increased by an average of 6% (*P* = 2.632e-05; *N* = 4 pairs of adult female Ts1Cje and disomic littermate controls; Figure 9B). There was no difference between the numbers of oligodendrocytes and unclassified cells produced by the Ts1Cje and disomic littermate control neurosphere cells (data not shown).

### Differentiated Ts1cje neurons have a tendency for reduced side branching

**Figure 10:**
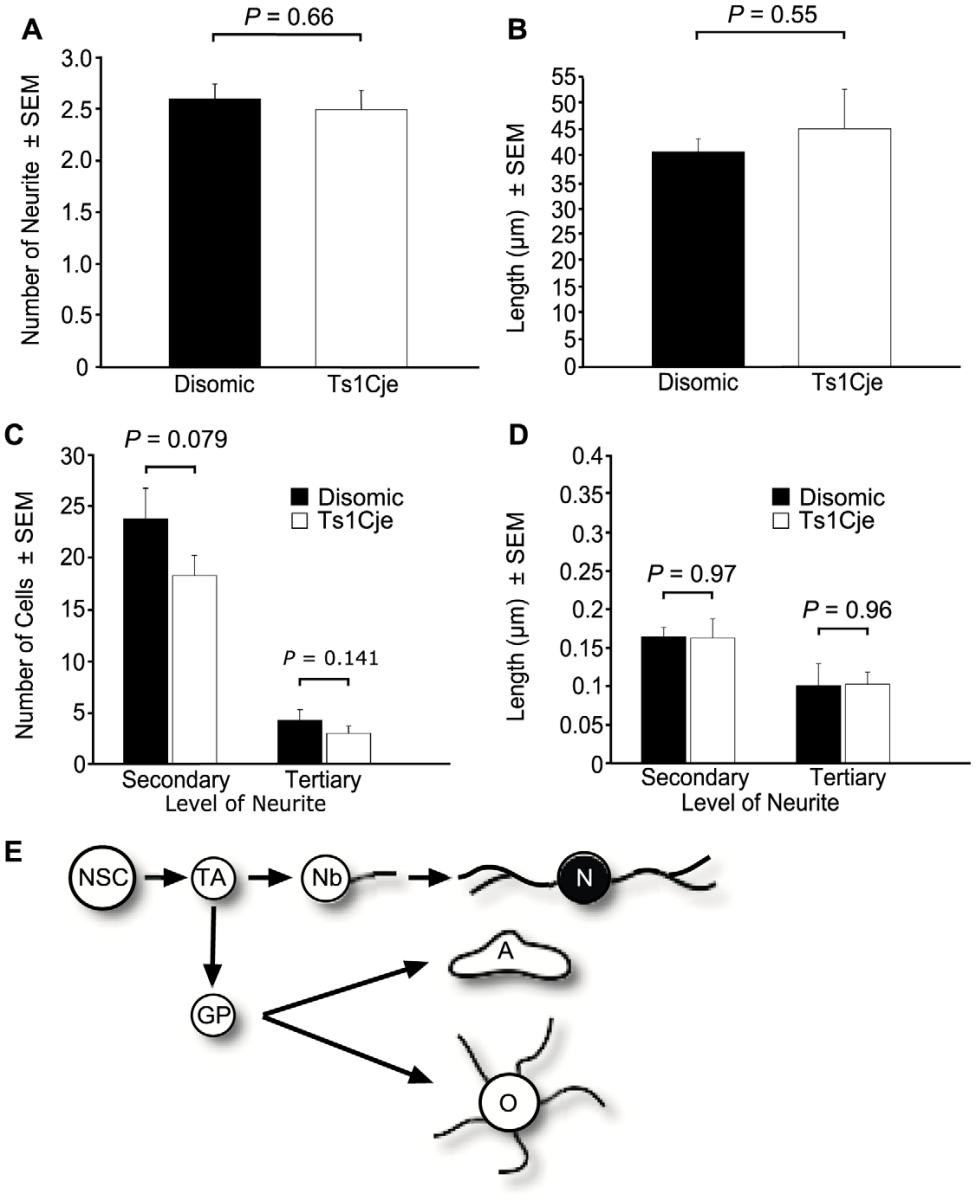
Assessment of neurite complexity. There was no difference in the number of primary neurites/cell (A) or length of primary neurite (*P* = 0.66) (B) between neurons differentiated from dissociated Ts1Cje and disomic neurosphere cultures (*P* = 0.55). (C) The number of Ts1Cje neurons with side branching was consistently reduced compared to disomic neurons. However, the difference was not statistically significant (secondary neurite *P* = 0.079; tertiary neurite *P* = 0.141), (D) there was no difference in their length (secondary neurite *P* = 0.97; tertiary neurite *P* = 0.96). *N* = 4 pairs of female Ts1Cje and disomic littermate controls. (E) Neural cell lineage schematic with the cell type examined highlighted – N, neuron. Other cells: NSC, neural stem cell; TA, transit amplifying cell; Nb, neuroblast; GP, glial precursor; A, astrocyte; O, oligodendrocyte.

Examination of the neurons that were produced by both genotypes showed that there was no difference in the number of primary neurites per cell (*P* = 0.66) or the average length (*P* = 0.55) of primary neurite (Figure 10A and B). The number of Ts1Cje neurons with a secondary or tertiary neurite was consistently lower than the number of disomic neurons. While the difference was not significantly statistically different (secondary neurite *P* = 0.079; tertiary neurite *P* = 0.141; *N* = 4 female Ts1Cje and sex matched disomic littermate controls; Figure 10C), lower neurite complexity has been observed in human DS and trisomic mice [67], [68], [69], [70], [71]. There was no difference in the average length of Ts1Cje and disomic secondary (*P* = 0.97) or tertiary (*P* = 0.96) neurites (Figure 10D).

## Discussion

We have used a combination of *in vivo* and *ex vivo* assays, a gene expression and network analysis to understand a number of aspects of adult neurogenesis in the Ts1Cje mouse model of DS. We have distinguished for the first time that reductions in cell numbers observed by others are due to reductions in neural progenitors and neuroblasts rather than NSCs in the Ts1Cje subventricular zone (SVZ). In this study we also observed dysregulated expression of genes involved in proliferation, particularly cell cycle control. We have shown a tendency for delayed neuroblast migration through the adult Ts1Cje SVZ. Importantly, we have uncovered severe defects in Ts1Cje neural differentiation, in particular a reduction in the production of neurons and a tendency for reduced side branching. Given that our work demonstrates neurogenesis defects exist during adulthood suggests that there is potential to slow aspects of the progression of cognitive decline and neurodegeneration in DS.

Analysis of our microarray expression data from adult SVZ neurosphere cultures using both the Database for Innovation, Visualisation and Integrated Discovery (DAVID) and Ingenuity Pathway Analysis (IPA) identified dysregulation of genes involved in proliferation in Ts1Cje samples. The majority of proliferation associated genes showing disrupted expression were subtly downregulated and not from the Ts1Cje trisomic region, indicating that trisomy results in a large number of downstream gene expression abnormalities that combined would affect proliferation in adult neural progenitors. The largest IPA network of differentially expressed genes included a number of genes whose down regulation could delay cell cycle progression. The following discussion is focused around the genes from this network for which expression was most affected by trisomy. The helicase minichromosome maintenance proteins (MCMs) and Prim1 (small subunit of primase, which synthesises oligoribonucleotides that act as primers for initiation of DNA synthesis) are involved in DNA replication and their reduced expression is likely to result in a delay or elongation of the S phase of the cell cycle [72], [73], [74]. Brca2 has various roles in the maintenance of genome stability, but its main function appears to involve the regulation of the function of Rad51 (DNA repair protein) during DNA repair by homologous recombination. Homologous recombination is the predominant mechanism of repair for double strand breaks during the S-phase and G_2_-phase of the cell cycle and Brca2 deficiency has been shown to impair cytokinesis and extend cell division [75], [76]. The CENP-H/I multiplex kinetochore complex, containing the centromere protein O (CENP-O; interphase centromere complex protein 36) has been shown to be required for proper chromosome alignment and segregation in chicken and human cells [77]. Furthermore, it has been demonstrated that CENP-O deficient cells had a lengthened G_2_/M phase [77]. Aurka is a member of a family of serine/threonine kinases and plays a critical role in mitosis. It is necessary for formation of mitotic spindles, separation of the centrosomes after the mitotic spindle has been formed and ensures correct organization and alignment of chromosomes during prometaphase [78], [79]. Therefore, its downregulation may prevent or slow exit from mitosis.

Based on the changes in gene expression that we observed we would expect a delay in cell cycle progression in the S or G_2_ and M phases. Indeed, elongation of the G_1_ but particularly the G_2_ phase has been observed in trisomic neural precursors [44], [47], [48]. Interestingly, it has been demonstrated that the expression of *Ccnb1* and *Skp2*, two key regulators of the G_2_/M and G_1_/S transitions are decreased in neonatal (PN2) Ts65Dn cerebellar granule cell precursors [47]. While these observations have been made in a different area of the brain and during development rather than in the adult, they support our data and suggest that delays in cell cycle transitions particularly the G_2_/M phase may be a common theme in trisomic cell division. Notably, we found *Ccnb1* to be downregulated in adult neurospheres. Furthermore, all probes located to the *Skp* loci on our microarray that were expressed in adult neurospheres were reduced in the Ts1Cje compared to disomic controls, with fold changes ranging from 0.64 to 0.87.

A recent gene expression study on embryonic neural progenitors cultured as neurospheres from the E14.5 Ts1Cje neocortex also observed dysregulation of genes involved in proliferation, although changes in expression of individual genes differed from our study [55]. These differences are likely to be due to a number of factors including the different age of mice that the neural progenitors were isolated from, the different microarray platforms used and the number of passages carried out prior to RNA extraction. Moldrich *et al*., (2009) used cells after at least four passages in culture, whilst we subjected cells to only one passage in culture before conducting the microarrays. We note that we have shown changes in gene expression in trisomic neural precursors even in different pathways to those observed by others despite some similarities in neural differentiation defects [80], [81]. In particular, we observed no deregulation in the expression of the neuron-restrictive silence factor (REST-repressor element-1 silencing transcription factor) or *Rest* regulated genes in the trisomic neural progenitor cells. This could be due to the differences in the source of cells investigated. We used primary neurospheres, whilst Bahn *et al*., (2002) analysed cells after more than 10 weeks of culture and Canzonetta *et al*., (2008) analysed cell lines following at least 3 passages. In this context it is important to note that striking differences have been observed between primary neuronal cells and cells cultured for extended periods such that primary cells are expected to reflect the situation *in vivo* more accurately [82], [83], [84].

To our knowledge, no previous study has differentiated between the effects of trisomy 21 in humans, or 16 in mice on NSCs versus neural progenitor cells. We did not detect a difference between the number of adult Ts1Cje and disomic control NSCs *in vivo* using a double thymidine analogue labelling assay. However, we observed a reduction in stem cell/progenitor derived primary neurospheres from the Ts1Cje SVZ and neuroblasts migrating through the RMS. A partial block in the S, G_2_ or M phases of the cell cycle could explain a low production of neural progenitors and neuroblasts. In concordance with previous findings and a reduction in the numbers of neural progenitors and neuroblasts the anterior-posterior length of the Ts1Cje olfactory bulbs were shorter than the disomic controls [54].

In support of our findings others have reported similar defects in the production of neural progenitors, albeit the majority of studies have been focused on different areas of the brain, at different stages of development. A number of studies of Ts16, Ts65Dn and DS embryonic brain development have identified cell cycle and neurogenesis abnormalities in the neocortex and hippocampus [44], [45], [49], [50], [51]. A substantial delay in prenatal growth of the Ts65Dn cortex and hippocampus was shown to be due to a longer cell cycle and reduced neurogenesis from the ventricular zone neural precursor population [44]. Correspondingly, reduction in the number of proliferating cells in the germinal zones of DS foetal hippocampus, parahippocampus and lateral ventricles has been demonstrated [48], [49]. Similarly, reduced cell proliferation has been demonstrated in the neocortical germinal matrix of human DS foetuses and the dentate gyrus of postnatal day 2 (PN2) and 6 Ts65Dn mice [41], [48]. Of the cells that were dividing in the DS foetal neocortex and Ts65Dn PN2 dentate gyrus, a higher proportion were in the G_2_ phase, which was also shown to be prolonged compared to the disomic [48]. Additionally, a reduction in the proliferative capacity due to increased cell cycle duration of neural progenitors cultured from embryonic neocortical Ts1Cje neurospheres has been described [55]. It has been demonstrated that postnatal development of the hippocampus is affected by abnormal proliferation in the subgranule zone [47], [48], [52]. Reduced proliferative capacity of the adult neural stem cell (NSC) population of the hippocampus has been shown in both young and old adult Ts65Dn mice [46], [53]. Recently, a reduction in the number of neuroblasts in the SVZ and a reduction in the number of proliferating cells was demonstrated in the SVZ of the lateral ventricles and dentate gyrus of adult Ts2Cje (genetically equivalent to Ts65Dn) and Ts1Cje [54]. The same authors also reported reduced cortical neurogenesis in the embryo of both mouse models.

Our neurosphere data imply that there is a major reduction in the capacity of Ts1Cje neural progenitors to differentiate into neurons by more than 50%. The number of neurons produced by the SVZ has not been assessed previously in models of DS. Defects in neural differentiation have been reported for other areas of trisomic brains and recently a greater number of GFAP-positive cells were observed prior to differentiation in neural progenitors isolated from the E14.5 Ts1Cje neocortex [46], [47], [48], [49], [55]. It has been suggested that the smaller number of neurons is attributable to the fact that precursors proliferate at a slower rate rather than, or in addition to, impaired differentiation. However, as our data of neuronal differentiation is based on equal numbers of neural progenitors, we observed a greater than 50% reduction in neurons produced and a compensating increase in astrocytes, we conclude that trisomic neural progenitors differentiate into astrocytes at the expense of neurons. This phenomenon constitutes a disturbance in cell lineage commitment and differentiation that cannot be explained solely by the reduction in progenitors, which we also observed.

We have demonstrated that the expression of a number of genes involved in control of the cell cycle are disrupted and that there are a number of abnormalities in adult Ts1Cje adult neurogenesis, however the question remains, which trisomic gene or genes are responsible? It is tempting to speculate that the abnormalities we have observed are due to the overexpression of the dual-specificity tyrosine-(Y)-phosphorylation regulated kinase 1A gene (*Dyrk1A*), which is located on the Ts1Cje trisomic region. Indeed, the 3 *Dyrk1A* probes on our microarray were overexpressed at expected trisomic levels. Dyrk1A has been shown to have a highly dynamic expression pattern throughout mouse brain development suggestive of a key role in regulating the multiple events that occur during neuronal development [85]. Expression during the S- and G_2_-phases of the neuronal progenitor cell cycle combined with the altered levels of cell cycle regulators found in mice overexpressing *Dyrk1A* and the phosphorylation of cell cycle regulators by DYRK1A led to the suggestion that it is a regulator of neural progenitor proliferation [85], [86], [87]. Dyrk1A is also co-expressed transiently in newborn neurons with high levels of p27^KIP1^, which has been shown to be involved in neuronal differentiation [85], [88], [89]. Transgenic mice overexpressing human or mouse *Dyrk1A* have been generated and were shown to display hippocampal-dependent spatial learning and memory defects, developmental delay and motor deficits [90]. Interestingly, it was recently shown that using shRNA to inhibit *Dyrk1A* in the striatum of a transgenic mouse model of *Dyrk1A* resulted in the restoration of motor coordination, attenuation of hyperactivity and improvement of sensorimotor gating [91].

Our data demonstrate the presence of neurogenesis defects in the adult trisomic brain at multiple levels in the neurogenic lineage, which we suggest will increase the vulnerability of DS neurons for neurodegeneration. We also hypothesise that the existence of adult nuerogenesis defects in the trisomic brain may offer the opportunity to develop therapies that might either slow down the progression or even improve the neuronal deficits. A number of recent studies have provided evidence to suggest that this is not an unreasonable suggestion, proposing that a number of agents can improve learning and memory and possibly even brain structure in the adult Ts65Dn mouse [92], [93], [94], [95].

In conclusion, we have investigated the characteristics of adult neurogenesis in the Ts1Cje brain and determined that; 1) Neurogenic cells isolated from the adult Ts1Cje brain exhibited disrupted expression of genes encoding proteins involved in cell cycle progression. 2) Ts1Cje brains had normal numbers of long-term retaining NSCs. 3) In contrast, the numbers of self-renewing neural progenitor colonies, were reduced. 4) There was a reduction in the number of neuroblasts migrating through the Ts1Cje RMS compared to disomic controls. 5) Our data suggests that the speed of adult Ts1Cje neuroblasts migration was reduced. 6) The number of neurons produced by adult Ts1Cje neurogenic progenitors showed a striking reduction to less than one half whilst the numbers of astrocytes was increased. 7) Ts1Cje neurons showed a tendency for less elaborate neurites.

## Materials and Methods

### Ethics Statement

All breeding and experiments were approved by the Walter and Eliza Hall Institute Animal Ethics Committee (Project numbers 2001.45, 2004.041 and 2007.007).

### Animals

Sex matched disomic littermates were used as controls for all experiments. All mice were aged to 3 months (84 days). Ts1Cje and disomic mice were generated by mating Ts1Cje males (originally obtained from The Jackson Laboratory, Bar Harbour, USA) with C57BL/6 female mice for over 10 generations. Offspring were genotyped using multiplex PCR primers for neomycin and *App* (as an internal control) as described previously [28] substituting gel electrophoresis with high resolution melting analysis [96].

### Microarray analysis

RNA was extracted from 3 Ts1Cje and 3 disomic female littermate control neurospheres (generated from adult subventricular zone (SVZ) cells) cultured for 7 days after isolation using a Qiagen RNeasy micro kit (Qiagen, Hilden, Germany) according to the manufacturer's instructions with the DNase I digestion step. The quality and quantity of RNA was assessed using a 2100 Bioanalyzer (Agilent Technologies, Santa Clara, USA). Each RNA sample was processed using the Two-Cycle Target Labelling Assay and hybridised onto an Affymetrix GeneChip® Mouse Genome 430 2.0 Arrays according to the manufacturer's instructions (Affymetrix, Santa Clara, USA). Fluorescent signals were detected using a GeneChip® Scanner 3000 (Affymetrix, Santa Clara, USA).

Expression data were pre-processed and normalized using the gcRMA algorithm [97]. Differential expression was assessed using empirical Bayes moderated *t*-statistics [98] and *P*-values were adjusted for multiple testing [99] using the limma package and R statistical software (www.r-project.org) [100]. An additional global analysis of the *P*-values was completed to estimate the true percentage of statistically significantly expressed genes [101]. The online analysis tools The Database for Annotation, Visualization and Integrated Discovery (DAVID; [61] and Ingenuity Pathway Analysis (IPA; from Ingenuity Systems Inc., Redwood City, USA; http://www.ingenuity.com) were used to uncover changes in functionally interconnected genes from a list of the most differentially expressed genes (Further description in Text S1). Using a *t*-statistic value of 4 as a cut off, probes that were over or underexpressed in the Ts1Cje neurospheres compared to disomic neurospheres were selected for analysis.

### Quantitative real time polymerase chain reaction (RT-qPCR)

RT-qPCR was used to validate the expression of 11 selected genes from the microarray analysis. RNA was extracted independently from the microarray samples from 3 female Ts1Cje and 3 disomic litermate control 7-day-old neurosphere culture pellets using a Qiagen RNeasy micro kit (Qiagen, Hilden, Germany) according to the manufacturer's instructions with the DNase digestion step. RNA quality and quantity was assessed using a 2100 Bioanalyzer (Agilent Technologies, Santa Clara, USA). First strand cDNA was synthesised using random hexamers and the SuperScript™III Reverse Transcriptase Kit (Invitrogen, Carlsbad, USA) according to the manufacturer's instructions. Primers were designed and probes selected using ProbeFinder Version 2.34 (Universal ProbeLibrary Assay Design Center, Roche Applied Science https://www.roche-applied-science.com). Primer and probe sequences are detailed in the Text S1. RT-qPCR was performed in triplicate on 330 ng total RNA template with 1X LC480 Master Probe Mix (Roche Diagnostics, Basel, Switzerland), 250 nM forward and reverse primers (GeneWorks, Thebarton, Australia), 100 mM Universal ProbeLibrary probe (Roche Diagnostics, Thebarton, Australia) made to a final volume of 10 µl with PCR-grade H_2_O (Roche Diagnostics, Thebarton, Australia), on a LightCycler® 480 System (Roche Diagnostics, Thebarton, Australia). Conditions for the RT-qPCR were a pre-denaturing step of 95°C for 10 min, 45 cycles of; 95°C for 10 sec, 60°C for 30 sec, 72°C for 30 sec and finishing with a cooling step at 40°C for 1 min.

The quantification cycle (Cq) for each signal was calculated based on the Second Derivative Maximum method. A set of serially diluted cDNAs synthesised from 3 µg of adult mouse brain RNA was used to construct a 3-data point standard curve for every PCR assay in each run. A PCR efficiency of between 90–110% and an R-squared value >0.98 were used to define successful assays. Relative quantification of target gene expression in Ts1Cje and disomic samples was carried out using the comparative Ct method. We performed intra-sample data normalization against 3 endogenous control reference genes *Pgk1*, *Hmbs* and *Psmb2*. Differential analysis was completed with the limma package [100] (R-script; Text S1) using empirical Bayes moderated *t*-statistics which borrows information between genes [98]. *P*-values were adjusted for multiple testing [99].

### Neurosphere proliferation and differentiation assays

Cells from the SVZ were isolated and cultured as described previously [56], [102], [103], [104], [105]. Briefly, avoiding white matter and using a dissection microscope (Stermi 2000-C, Zeiss, Oberkochen, Germany), the SVZ of the lateral walls of the lateral ventricles were bilaterally dissected. The dissected tissue was immersed in 2.5% pancreatin/0.5% trypsin in D-PBS (Ca^2+^/Mg^2+^ free) with 200 U/ml penicillin, 200 µg/ml streptomycin for 30 min at 4°C and transferred into mouse tonicity phosphate-buffered saline (MTPBS) for 5 min at 37°C. The pancreatin/trypsin was inhibited briefly by 2% foetal bovine serum in MTPBS, and the tissue was mechanically dissociated. Cells were collected by centrifugation and cultured in neural stem cell (NSC) proliferation medium for 7 days: DMEM/F12, 5 mM HEPES, 13.4 mM NaHCO_3_, 0.6% D-glucose, 100 U/ml penicillin, 100 µg/ml streptomycin, 25 µg/ml insulin, 60 µM putrescine dihydrocloride, 100 µg/ml apotransferrin, 30 nM sodium selenite, 20 nM progesterone, 10 ng/ml bovine recombinant fibroblast growth factor 2 (FGF2), 4 µg/ml porcine heparin sodium salt, 20 ng/ml murine epidermal growth factor (EGF) and 2 mg/ml bovine serum albumin fraction V. Under these conditions SVZ cells proliferate and form neurospheres. Neurospheres were collected by centrifugation and dissociated in 0.25 mg/ml trypsin, 10 µg/ml DNase I, 10 mM HEPES and 0.2 mg/ml EDTA in Ca^2+^/Mg^2+^ free HBSS at 37°C for 3 min. The trypsin was inhibited briefly by 140 µg/ml soybean trypsin inhibitor, in HEM with 10 µg/ml DNase I. 50,000 cells were plated onto poly-ornithine/laminin-coated 13 mm glass coverslips in differentiation medium (proliferation media excluding FGF2, EGF and heparin sodium salt with 1% bovine serum albumin fraction V) for 8 days.

### Thymidine analogue treatment strategies and analysis

For assessment of neuroblast migration along the rostral migratory stream (RMS) 4 pairs of Ts1Cje and disomic littermate controls (3 male and 1 female pairs) were injected with 5-bromo-2′deoxyuridine (BrdU; 100 µg/g i.p.) twice daily 12 h apart for 4 days and left without treatment for 2 days. The migration front of BrdU labelled neuroblasts was identified on serial paraffin sections stained for BrdU. Adjacent sections were stained with cresyl violet to reveal histological morphology. To determine the distance migrated by the neuroblasts, the distance between the most rostral extent of the frontal cortex, as a reference point and the migration front was calculated based on the number of intervening sections and section thickness.

To determine the number of NSCs and progenitors and to assess their proliferation 4 pairs of male Ts1Cje and disomic littermate controls were injected with 5-iodo-2′-deoxyuridine (IdU; 100 µg/g i.p.) twice daily 12 h apart for 7 days, left without treatment for 7 days and then injected with 5-chloro-2′-deoxyuridine (CldU; 100 µg/g i.p.) twice daily 12 h apart for 7 days and finally left without treatment for 7 days. The number of CldU positive, IdU positive and double positive cells were counted at five equally spaced rostrocaudal levels approximately 250 µm apart ranging from the most rostral section containing fimbria of the hippocampi to the section containing the fusion of the rhinal fissure. Stained sections were analysed microscopically and photographs were taken with a digital camera (Stemi 2000-C, dissection microscope and Zeiss Axiocam HRc software Axiovision, Zeiss, Oberkochen, Germany).

### Immunohistochemistry and Immunofluorescence

To ensure that assays involving cell counts were accurate, the nuclei of cells throughout the SVZ from digital images at a magnification of 40× of 4 pairs of sex matched adult Ts1Cje and disomic littermates were compared. The longest diameter of 630 randomly selected nuclei from each genotype were measured. We found no significant difference in the mean length of the longest nuclei diameter between Ts1Cje and disomic controls (data not shown) and therefore numbers of labelled SVZ cell profiles could be compared [106]. A general anatomical analysis of the whole brain was completed to assess whether there are any gross structural abnormalities in the Ts1Cje brain (for methods and results refer to Text S1).

For thymidine analogue incorporation studies, brains were perfusion-fixed in 4% PFA, postfixed in 4% PFA for 48 h, embedded in paraffin and coronally sectioned at 5 µm in preparation for staining.

Immunohistochemistry was performed as described previously [107] using an antibody against BrdU (M0744 Clone Bu20a; 1∶10; Dakocytomation, Glostrup, Denmark). Immunofluorescence was performed on brain sections [108] and cells [109] as described previously. Cells were incubated with antibodies against β-tubulin type III (G7121; 1∶2000; Promega, Madison, USA), and GFAP (MAB3402; 1∶1000; Chemicon) and sections were incubated with antibodies against BrdU that also detects CldU but not IdU (OBT-0030; clone BU1/75; 1∶100; Accurate Chemical & Scientific Corporation, Westbury, USA), all diluted in 10% goats serum. Cells and sections were subsequently incubated with appropriate fluorescently label-conjugated secondary antibodies (Molecular Probes, Invitrogen, Carlsbad, USA; Vector laboratories, Burlingame, USA). An antibody against BrdU (clone B44; 1∶3.5; BD Biosciences, San Jose, USA), which also detects IdU but not CldU was conjugated with FITC and was therefore added to the sections with the secondary antibodies. Nuclei were counterstained with bisBenzimide H 33258 (Sigma-Aldrich®, St Louis, USA) or DAPI (4′,6-diamidino-2-phenylindole; Vector Laboratories, Burlingame, USA). Anti-O4 (1∶10; gift from T. Kilpatrick Howard Florey Institute, Parkville, Australia) primary incubation was performed without any prior permeabilisation.

Four pairs of sex matched (2 female, 2 male) Ts1Cje and disomic littermate controls were assessed for the number of apoptotic cells in the SVZ using the ApopTag® Fluorescein *In Situ* Apoptosis Detection Kit S7110 according to the manufacturer's instructions (Chemicon International, Temecula, USA). The number of TUNEL positive cells was counted at 5 evenly spaced rostrocaudal intervals on sections adjacent to those assessed for thymidine analogue incorporation.

Whole-mount immunohistochemistry was performed as described previously [102], [110] on 5 pairs of Ts1Cje and disomic littermate controls (2 male and 3 female). Briefly, the entire lateral walls of the lateral ventricles were dissected from perfusion-fixed (3% PFA) brains and were immersed in cold methanol, cold acetone, 10% goats serum, incubated with an anti-polysialic acid-neural cell adhesion molecule (PSA-NCAM) antibody (AbC0019; 1∶1000; AbCys, Paris, France) for 72 h at 4°C, and then with a biotinylated secondary antibody for 48 h at 4°C, endogenous peroxidase activity was quenched with 0.3% H_2_O_2_ in methanol, and bound antibody was visualised using horseradish peroxidase-coupled streptavidin, diaminobenzidine and H_2_O_2_. Intervening wash steps were TBS with or without 0.5% Triton X-100. Photographs of stained tissue were taken using a low magnification stereomicroscope (Stemi 2000-C, Zeiss, Oberkochen, Germany) and digital camera (AxioCam, Zeiss, Oberkochen, Germany), PSA-NCAM-positive chains were traced and quantified using the NIH Image analysis software, ImageJ.

### Statistical analysis

Stata®10 (StataCorp LP, Texas, USA) software and R language and environment were used to perform multifactorial ANOVAs and other statistical analyses (for further details see Text S1).

## Supporting Information

Text S1 Supporting Text.
(0.13 MB DOC)

Table S1 DAVID data.
(0.21 MB XLS)

Table S2 Affymetrix and RT-qPCR data.
(0.03 MB XLS)
