# Supplementary material for: Gene Network Disruptions and Neurogenesis Defects in the Adult Ts1Cje Mouse Model of Down Syndrome
Source: PLoS One. 2010 Jul 16;5(7):e11561. doi: 10.1371/journal.pone.0011561 (PMC2905390; doi:10.1371/journal.pone.0011561)
Supplement: Text S1 — Supporting Text. (0.13 MB DOC) [file pone.0011561.s001.doc]

**Supporting Text For Gene Network Disruptions and Neurogenesis Defects in the Adult Ts1Cje Mouse Model of Down Syndrome**

**Supporting Introduction text**

**Summary of Down syndrome mouse models**

Down syndrome mouse models trisomic for all or part of MMU16 or HSA21, adapted from Patterson (2009).

| **Name** | **Number of trisomic genes** | **Mb of trisomic DNA** | **Trisomic region** | **References** |
| --- | --- | --- | --- | --- |
| Ts16 | ~731 | 92.8 | MMU16 | [1,2] |
| Dp(16)1Yu/p | ~113 | 22.9 | MMU16 from D930038D033Rik-telomere | [3] |
| Ts65Dn | ~94 | 13.7 | MMU16 from Mro139-telomere | [3,4] |
| Ts2Cje | ~94 | 13.7 | Genetically equivalent to Ts65Dn; Ts65Dn marker chromosome translocated to MMU12 | [5] |
| Ts1Cje | ~70 | 8.0 | MMU16 from SOD1 (not functional)-telomere | [6] |
| Ts1Rhr | ~33 | 4.2 | MMU16 from Mx1-Cbr1 | [7] |
| Tc1 | ~146 | ~42 | Contains most of HSA21 | [8] |

**Supporting Results**

We completed a general anatomical analysis of the whole brain to assess whether the abnormalities we identified produce any gross structural differences.

**Ts1Cje mice have small paraflocculi**

We observed no difference in mouse weight, brain weight or brain weight to body weight ratio between adult Ts1Cje and sex matched disomic littermate controls (n = 5 pairs; data not shown). The brains of two littermate pairs of adult female Ts1Cje and disomic controls were stained with cresyl violet following cardiac perfusion with PBS and 4% PFA. The only obvious difference in brain histology observed was that Ts1Cje mice had smaller paraflocculi than their disomic littermates (Data not shown).

The floculus/paraflocculus system is involved in control of eye movement and deficiencies in this system can result in nystagmus [9,10,11]. Ocular abnormalities including nystagmus in persons with DS are common and have been reported with varying frequencies [12,13,14]. It is possible that a reduction in the size of the paraflocculus could be involved in the nystagmus and ocular abnormalities observed in DS.

**Supporting Material and Methods**

***Dnahc11***

The Ts1Cje mutation was generated during the targeted mutation of *Sod1* due to the translocation of distal chromosome 16 onto the very distal end of chromosome 12, two wildtype copies of chromosome 16 were recovered by breeding with wildtype mice [6,15]. *Dnahc11* (Dynein axonemal heavy chain 11) was most the statistically significant gene (adjusted p value <0.05) in the microarray profiling between adult Ts1Cje and disomic neurospheres, but we do not believe it to be relevant to DS. It is situated on the distal end of chromosome 12, very close to the translocation break point and it is therefore likely that the increased expression of *Dnahc11* is specific to the Ts1Cje model and therefore not relevant to DS. Indeed, *Dnahc11* has not been found to be overexpressed in the majority of other global gene expression investigations of the DS or DS mouse models brain [16,17,18,19,20,21]. However, recently, Laffaire *et al*., (2009) demonstrated that a 2Mb segment of MMU12 including the *Dnahc11* locus is deleted in the Ts1Cje model, which would suggest that its expression should be reduced. However, in contradiction to this and similar to our results Laffaire *et al*., (2009) report that the expression of *Dnahc11* was increased in the post-natal developing Ts1Cje cerebellum.

**DAVID and IPA**

DAVID provides a comprehensive set of functional annotation tools to help find biological meaning behind large gene lists mainly using standardised Gene Ontology terms [22]. In contrast, IPA identifies associations that exist between genes using the Ingenuity Pathways knowledge base, which is based on current literature and online databases to build up networks or pathways.

**RT-qPCR primer and probe details**

Primers were designed and probes selected using ProbeFinder Version 2.34 (Universal ProbeLibrary Assay Design Center, Roche Applied Science [https://www.roche-applied-science.com](https://www.roche-applied-science.com/)) with the following parameters for primer design; primer length 18-27bp, GC content 30-70%, T_m_ 59-60^o^C and amplicon size 50-250bp. Primers were manufactured by GeneWorks (Thebarton, Australia)

| **Gene** | **Primer sequence (F/R)** | **Amplicon size (bp)** | **UPL probe** |
| --- | --- | --- | --- |
| Brca2 | gctacaccaccaacccttagtt / agagctgcgtgcttcttcat | 63 | 99 |
| Mcm7 | cccgacagagacaatgacct / gcacatggctatgtaccgtct | 126 | 107 |
| Phb | cacggaacgtaccagtcatc / cgccaatgctggtgtagata | 117 | 20 |
| Ccna2 | tctacacagtcacaggacagagctggc / gtgaaggtccacaagacaaggctt | 95 | 80 |
| Aurka | tttgacgagcagagaacagc / ccgtttgagccaagcagta | 122 | 106 |
| Suv39h1 | ttgttatggagtatgtgggagaga / tccacggtatatacgtcttcca | 127 | 85 |
| Pbk | cttccagatgatgatgttgatga / agagttcaatggccttctgg | 142 | 42 |
| Cenpo | tgggaatacctaaatgcctacg / aggacatcgcagaagtcactc | 74 | 69 |
| Ccnb1 | gaaccagaggtggaacttgc / ggcttggagagggattatca | 109 | 2 |
| Itsn1 | aaggcacaatcattcgatgtc / tttcagccttgatgactgagg | 75 | 67 |
| Prim1 | aggcgcagtatattctcacaga / catgtcgatgtcaaagaccagt | 97 | 2 |
| Pgk1 | tacctgctggctggatgg / cacagcctcggcatatttct | 65 | 108 |
| Psmb2 | gagggcagtggagcttctta / aggtgggcagattcaagatg | 71 | 25 |
| Hmbs | aaagttccccaacctggaat / ccaggacaatggcactgaat | 98 | 42 |

**R-script for RT-qPCR differential analysis**

> library(limma)

> dataFilename <- "data_file.txt"

> ratios <- read.delim(file=dataFilename, row.names=1)

> tissue <- factor(c("Ts1","Ts1","Ts1","WT","WT","WT"), levels=c("WT","Ts1"))

> mm <- model.matrix(~tissue)

> fit <- lmFit(ratios, design=mm)

> fit2 <- eBayes(fit)

> ttallc <- topTable(fit2, coef=2, number=Inf, sort="none")

**Histological analysis**

Two pairs of female Ts1Cje and disomic littermate controls were cardiac perfusion-fixed with 4% paraformaldehyde (PFA), postfixed in 4% PFA for 48 hours, embedded in paraffin and coronally sectioned at 5μm, stained with cresyl violet and analysed microscopically, photos were taken with a digital camera (Zeiss dissection microscope and Zeiss Axiocam HRc software Axiovision, Zeiss, Oberkochen, Germany).

**Statistical analysis**

For the adult neurosphere data, linear models with effects for genotype (Ts1Cje/disomic control) and sex (male/female) were fitted separately to the cell sizes and the square-root transformed neurosphere counts. ANOVA F-tests were used to assess differences attributable to genotype.

For the neurosphere differentiation data, generalised linear models with effects for day of collection and genotype were fitted to the counts for each cell type (neurons, astrocytes and oligodendrocytes). A logit link function was used along with a quasi-binomial approximation to the likelihood to account for over-dispersion. Analysis of deviance was used to assess the relative importance of genotype in each model.

For the neurite branching data, linear models with effects for mouse-pair and genotype were fitted to secondary and tertiary branch numbers. ANOVA was used to assess the importance of genotype.

**Supporting References**

1. Miyabara S, Gropp A, Winking H (1982) Trisomy 16 in the mouse fetus associated with generalized edema and cardiovascular and urinary tract anomalies. Teratology 25: 369-380.

2. Mural RJ, Adams MD, Myers EW, Smith HO, Miklos GL, et al. (2002) A comparison of whole-genome shotgun-derived mouse chromosome 16 and the human genome. Science 296: 1661-1671.

3. Li Z, Yu T, Morishima M, Pao A, LaDuca J, et al. (2007) Duplication of the entire 22.9 Mb human chromosome 21 syntenic region on mouse chromosome 16 causes cardiovascular and gastrointestinal abnormalities. Hum Mol Genet 16: 1359-1366.

4. Davisson MT, Schmidt C, Akeson EC (1990) Segmental Trisomy of murine chromosome 16: anew model system for studying Down syndrome. In: Patterson D EC, editor. Molecular genetics of chromosome 21 and Down syndrome. New York: WIley. pp. 263-280.

5. Villar AJ, Belichenko PV, Gillespie AM, Kozy HM, Mobley WC, et al. (2005) Identification and characterization of a new Down syndrome model, Ts[Rb(12.1716)]2Cje, resulting from a spontaneous Robertsonian fusion between T(171)65Dn and mouse chromosome 12. Mamm Genome 16: 79-90.

6. Sago H, Carlson EJ, Smith DJ, Kilbridge J, Rubin EM, et al. (1998) Ts1Cje, a partial trisomy 16 mouse model for Down syndrome, exhibits learning and behavioral abnormalities. Proc Natl Acad Sci U S A 95: 6256-6261.

7. Olson LE, Roper RJ, Sengstaken CL, Peterson EA, Aquino V, et al. (2007) Trisomy for the Down syndrome 'critical region' is necessary but not sufficient for brain phenotypes of trisomic mice. Hum Mol Genet 16: 774-782.

8. O'Doherty A, Ruf S, Mulligan C, Hildreth V, Errington ML, et al. (2005) An aneuploid mouse strain carrying human chromosome 21 with Down syndrome phenotypes. Science 309: 2033-2037.

9. Dieterich M, Brandt T (2008) Functional brain imaging of peripheral and central vestibular disorders. Brain 131: 2538-2552.

10. Rambold H, Churchland A, Selig Y, Jasmin L, Lisberger SG (2002) Partial ablations of the flocculus and ventral paraflocculus in monkeys cause linked deficits in smooth pursuit eye movements and adaptive modification of the VOR. J Neurophysiol 87: 912-924.

11. Zee DS, Yamazaki A, Butler PH, Gucer G (1981) Effects of ablation of flocculus and paraflocculus of eye movements in primate. J Neurophysiol 46: 878-899.

12. Catalano RA (1992) Opthalmologic concerns. In: Pueschel S, Pueschel J, editors. Biomedical concerns in persons with Down syndrome. Baltimore: Paul H Brookes Publishing Co. pp. 59-68.

13. Fimiani F, Iovine A, Carelli R, Pansini M, Sebastio G, et al. (2007) Incidence of ocular pathologies in Italian children with Down syndrome. Eur J Ophthalmol 17: 817-822.

14. Stephen E, Dickson J, Kindley AD, Scott CC, Charleton PM (2007) Surveillance of vision and ocular disorders in children with Down syndrome. Dev Med Child Neurol 49: 513-515.

15. Huang TT, Yasunami M, Carlson EJ, Gillespie AM, Reaume AG, et al. (1997) Superoxide-mediated cytotoxicity in superoxide dismutase-deficient fetal fibroblasts. Arch Biochem Biophys 344: 424-432.

16. Amano K, Sago H, Uchikawa C, Suzuki T, Kotliarova SE, et al. (2004) Dosage-dependent over-expression of genes in the trisomic region of Ts1Cje mouse model for Down syndrome. Hum Mol Genet 13: 1333-1340.

17. Contestabile A, Fila T, Bartesaghi R, Ciani E (2008) Cell Cycle Elongation Impairs Proliferation of Cerebellar Granule Cell Precursors in the Ts65Dn Mouse, an Animal Model for Down Syndrome. Brain Pathol 19: 224-237.

18. Kahlem P, Sultan M, Herwig R, Steinfath M, Balzereit D, et al. (2004) Transcript level alterations reflect gene dosage effects across multiple tissues in a mouse model of down syndrome. Genome Res 14: 1258-1267.

19. Mao R, Zielke CL, Zielke HR, Pevsner J (2003) Global up-regulation of chromosome 21 gene expression in the developing Down syndrome brain. Genomics 81: 457-467.

20. Saran NG, Pletcher MT, Natale JE, Cheng Y, Reeves RH (2003) Global disruption of the cerebellar transcriptome in a Down syndrome mouse model. Hum Mol Genet 12: 2013-2019.

21. Laffaire J, Rivals I, Dauphinot L, Pasteau F, Wehrle R, et al. (2009) Gene expression signature of cerebellar hypoplasia in a mouse model of Down syndrome during postnatal development. BMC Genomics 10: 138.

22. Ashburner M, Ball CA, Blake JA, Botstein D, Butler H, et al. (2000) Gene ontology: tool for the unification of biology. The Gene Ontology Consortium. Nat Genet 25: 25-29.
